# Qualitative study on diabetic cutaneous wound healing with radiation crosslinked bilayer collagen scaffold in rat model

**DOI:** 10.1038/s41598-023-33372-z

**Published:** 2023-04-19

**Authors:** Hongwei Li, Xin Chen, Kang Ren, Lihao Wu, Gong Chen, Ling Xu

**Affiliations:** 1 State Key Laboratory of Molecular Vaccinology and Molecular Diagnostics, Department of Laboratory Medicine, School of Public Heath, Xiamen University, Xiamen, 361102 People’s Republic of China; 2 Department of Burn, Beijing Jishuitan Hospital, Beijing, 100035 People’s Republic of China; 3 Shenzhen Research Institute of Xiamen University, Shenzhen, 51800 People’s Republic of China

**Keywords:** Biochemistry, Materials science

## Abstract

Diabetes may leave patients more prone to skin problems, and minor skin conditions can more easily turn into serious damage to the extracellular matrix, which further impairs the skin's mechanical properties and delays wound healing. Therefore, the aim of the work is to develop extracellular matrix substitution to remodel the mechanical properties of diabetic cutaneous wound and thus accelerate diabetic wound healing. A green fabrication approach was used to prepare radiation crosslinked bilayer collagen scaffold from collagen dispersion. The morphological, mechanical and swelling characteristics of radiation crosslinked bilayer collagen scaffold were assessed to be suitable for cutaneous wound remodeling*.* The feasibility of radiation crosslinked bilayer collagen scaffold was performed on full-skin defect of streptozotocin-induced diabetic rats. The tissue specimens were harvested after 7, 14, and 21 days. Histopathological analysis showed that radiation crosslinked bilayer collagen scaffold has beneficial effects on inducing skin regeneration and remodeling in diabetic rats. In addition, immunohistochemical staining further revealed that the radiation crosslinked bilayer collagen scaffold could not only significantly accelerate the diabetic wound healing, but also promote angiogenesis factor (CD31) production. Vascularization was observed as early as day 7. The work expands the therapeutic ideas for cutaneous wound healing in diabetes.

## Introduction

Approximately 537 million adults are living with diabetes^1–3^. Unlike normal wound healing, diabetic wound healing process undergoes prolonged inflammation, insufficient angiogenesis, and ultimately delays healing^3–5^. The impaired healing process breaks the balance of extracellular matrix (ECM) remodeling. It leads to a reduction in ECM deposition, especially collagen content. This makes inferior mechanical properties of the diabetic wound. However, the ECM, as cell interactive scaffold, provides mechanical support for adhesion, proliferation, and migration of various cells. Therefore, it is vital to develop ECM-like/collagen substitute to remodel the mechanical properties of the wound and thus accelerate diabetic wound healing^6^.

Mimicking the ECM structure and inducing angiogenesis are critical to the success of wound healing. Angiogenic molecules, such as growth factor, biological macromolecules and metallic ions, are introduced into scaffold^7^. However, scaffolds containing angiogenic molecules still have some disadvantages and limit their applications. Some studies have shown that the neoplastic potency of certain genes may be enhanced when growth factors and metallic ions are present^8–10^. Among them, one of the most important shortcomings is that the activity and release rate of those angiogenic molecules need to be controlled precisely. If not, it can lead to the loss of molecule activity and effect, mismatch between the release rate and wound healing rate, and massive release of local molecules leading to adverse reactions. It can hinder the formation of new blood vessels and delay wound healing. Therefor It is of vital practical significance to focus on biomaterials. More critically, matrices (or engineered biomaterials), rather than the biochemical molecules, play pivotal roles in stimulating angiogenic processes^7^. And changes in scaffold architecture can modulate angiogenesis without the need for complex exogenous growth factors^11^.

Collagen type I is the most prevalent component of the ECM and an ideal biomaterial for cutaneous substitute preparation^12–14^. Collagen type I is a purified product already used in medical devices as skin substitutes, which are approved by Food and Drug Administration (FDA) -approved^15^. For an abundant yet sustainable and cost-effective supply of collagen type I, studies found that crosslinked collagen are able to improve wound healing by deposition of oriented collagen and fast re-epithelialization^14,16,17^. However, natural collagen type I has some limitations in hydrogel/scaffold preparation, mainly for poor mechanical strength, thermal stability, and enzyme resistance^16,18^. Radiation has demonstrated the ability to modify collagen's molecular structure to minimize degradation and enhance mechanical stability^16^. What’s more, the collagen scaffold synthesized by radiation shows the ability to promote cell cycle from G_0_/G_1_ phase to S phase, and enhanced deoxyribonucleic acid synthesis to accelerate cell proliferation^19,20^. Thus, radiation synthesized collagen scaffolds are one of the most essential biomaterials for wound healing. Green radiation technology not only ensures the purity of the collagen material, but can also occur at room temperature. Therefore, it would be desirable to develop radiation crosslinked dermal scaffold from bovine collagen without cells for diabetic wound healing.

Using collagen type I and radiation crosslinking technology, a product of pure collagen with silicone membrane called radiation crosslinked bilayer collagen scaffold (rcBCS) was created. The rcBCS was designed as bilayer membrane system to be applied following diabetic skin defects to viable tissue. The dermal substitute layer in rcBCS was made of a three-dimensional porous matrix of collagen type I with radiation crosslinking technology. The temporary epidermal substitute layer in rcBCS was made of synthetic silicone and functions to manage moisture loss and prevent bacteria. Contrary to existing skin substitutes, it serves as a template for dermis reconstruction without the need for epidermal grafts. Compared to other products that also contain no cells, no cross-linking agent is required in the preparation process, making the product pure. Thus, the characteristics of collagen can be utilized extensively. The physico-chemical properties of rcBCS were assessed to verify the ability to remodel the mechanical properties of diabetic cutaneous wound, providing mechanical support and inducing angiogenesis as a temporary and transitional ECM/collagen substitute. After establishing the full-thickness diabetic cutaneous wound model in rats, the effects of inducing modulation of inflammatory response, angiogenesis and tissue remodeling were assessed. This study further explored the feasibility of rcBCS in clinical translation, giving the study practical significance with the simple and green technique and without the need for human donor tissue or tissue banking infrastructure.

## Methods

### Materials

Bovine collagen type I was the gift to obtained from Wuxi Biot Bio-technology Co., Ltd. (China), which was isolated from fresh bovine tendons and commercially available. Silicone membrane and liquid silicone were fabricated by medical grade synthetic polysiloxane polymer from Tianling Guijiao Co., Ltd. (China). The secondary dressing (Tegaderm™ Film) was purchased from Minnesota Mining and Manufacturing Company (3M, USA). All other reagents were analytical grade and used as received. Milli Q water was used throughout the experiments (RODI Laboratory Water Purification System, Xiamen RSJ Water Purification Technology Co.,Ltd, China).

### Preparation of radiation crosslinked bilayer collagen scaffold

The rcBCS consisted of collagen scaffold and silicone membrane. Collagen scaffold was prepared as previously described with some modifications^19^. Briefly, bovine collagen type I and Milli Q water were mixed by an ARE-310 hybrid mixer (Thinky, Japan) to make collagen dispersion with a total concentration of 0.5% (w/v). The collagen dispersion was crosslinked by electron beam radiation (Xiamen Jinri Pharmaceutical Co., Ltd., China) at room temperature to form collagen hydrogel. And the radiation exposure dose was 5 k Gy. Then collagen hydrogel was frozen at − 20 °C overnight and procedural vacuum freeze-dried (Beijing Songyuanhuaxing Technology Develop Co., Ltd., China) for 48 h to obtain collagen scaffold. Liquid silicone acted as an adhesive, and it spread homogeneously over the silicone membrane. The collagen scaffold and silicone membrane were integrated with liquid silicone to form bilayer collagen scaffold (BCS). Finally, the rcBCS was packaged in a sterilization package and then sterilized by electron beam radiation. (Supplementary Fig. 1 provides information on packaging and sterilization procedures.)

### Physico-chemical characterization of radiation crosslinked bilayer collagen scaffold

#### Morphology analysis

The samples were sputter-coated (Jeol, JFC-1600, Japan) with a thin layer of gold approximately 6 nm for 60 s with sputter current of 30 mA. Digital morphology images of rcBCS were obtained by field emission environmental scanning electron microscopy (FE-ESEM, FEI Quanta 650 FEG, USA) at an accelerated voltage of 30 kV with XT Microscope Control software (version 6.28, FEI Quanta 650 FEG, USA). Following digital image acquisition, membrane thicknesses and pore sizes were measured the software described above.

#### Mechanical properties in vitro

Collagen scaffold and rcBCS with cube shape (20 mm in length, 20 mm in width and 2 mm in height) were prepared for compression and tension testing. At least three samples were prepared for one condition. TA.XTplus100C texture analyzer (Stable Micro Systems, UK) was used to perform compression and tension testing. In the tension test, the samples were fixed to the tensile grip (A/TG), and the samples were pulled at a rate of 0.2 mm/s until the samples ruptured. The compression test was performed using a 36 mm diameter round probe (P/36R). The speed of the probe was 0.2 mm/s, and measurements were made at 200% relative deformation (the probe touches the sample and compresses down 4 mm)^21^. All data, including ultimate strength and characteristic parameters such as stress–strain at maximum load and energy at maximum load, were processed and obtained by Texture Analysis software (Stable Micro Systems, UK).

#### Swelling ratio

The collagen scaffold and rcBCS were weighed and rehydrated to measure the swelling ratio. The wet weight was measured at several points in time after rehydration. The sample swelling ratio of samples was calculated using the following equation

$$ {\text{Swelling ratio}}\left( \% \right) = \left( {{\text{W}}_{s} {-}{\text{W}}_{i} } \right)/{\text{W}}_{i} \times {1}00\% , $$

where W_*s*_ indicates the weight of the swollen collagen scaffold or rcBCS at each time point and W_*i*_ represents the weight of the collagen scaffold or rcBCS.

### Tissue modeling evaluation in vivo

#### Laboratory animals

Specific Pathogen Free (SPF) level Sprague–Dawley (SD) rats (male, 180 g-200) were chosen for this study, and obtained from Xiamen University Laboratory Animal Center. All protocols were performed in the same facility which maintained under standard light–dark cycle (12:12 h) at ambient temperature (23 ± 2 °C) and humidity (55 ± 10%). The living conditions and experimental procedures comply with the guide for the care and use of laboratory animals (National Institutes of Health)^22^. For one week before the initial procedure, rats were allowed to acclimate.

#### Establishment of a diabetic rat and randomization schedule

SD rats were fed a high-fat diet (> 20% fat, > 20% sugar, > 1% cholesterol, > 0.2% bile salts, Nanjing Shengmin Animal Farm Co., Ltd., China) for four weeks. Then, each SD rat received a single intraperitoneal injection of freshly dissolved streptozotocin (STZ, 40 mg/kg, Sigma) in sterile water. After one week, rats with two random fasting blood glucose levels > 16.7 mmol/L were selected as diabetic rats.

Diabetic rats were designated randomly as either rcBCS group or sham operation group by coin toss. Previous studies served as a reference in determining sample size^14,23^. In each group, there were at least twelve study sites available at any given time (Supplementary Table 1).

#### Diabetic cutaneous wound model

Diabetic rats were anesthetized by intraperitoneal injection of 1% (w/w) pentobarbital solution (4 mg/100 g) and sterilized with 5% povidone-iodine^24^. Four full-skin defect wounds were created symmetrically on the mid-dorsum with surgical scissors. While the sham operation group had wound sizes of roughly 1.75 cm × 1.75 cm, the rcBCS group had wound sizes of about 2 cm × 2 cm. The skin defect wound in rcBCS group was covered with rcBCS and then sutured. The rcBCS served as temporary skin substitute and was not changed during the study. Gradually, it degrades to make room for dermal regeneration. The phenomenon of wound contraction has been studied in the skin of rats^25^. To avoid the confusing bias brought on by wound contraction, Tegaderm™ Film (3M, USA) was utilized as an outer dressing. The Tegaderm™ Film was sticky and played a role in fixing the position of the skin around the wound to avoid inward contraction. In contrast, no graft materials but only Tegaderm™ Films were placed in sham operation group.

#### General observation of the wound

At predetermined postoperative time points (days 0, 3, 7, 14 and 21), macroscopic images of the wounds were recorded with a digital camera (Meizu, Zhuhai, China). An excision wound margin was tracked after wound creation using Photoshop (Adobe systems incorporated, USA) and area was measured.

#### Histological study

Diabetic rats were sacrificed and regenerated tissue specimens from the wound area were harvested at predetermined time points (days 0, 7, 14 and 21). Regenerated tissue specimens were fixed in 10% neutral buffered formalin, then dehydrated in ethanol and embedded in paraffin. Sections of 5 μm were prepared. For sections, hematoxylin–eosin (H&E) and Masson’s trichrome staining were used. The procedures of H&E and Masson’s trichrome staining were described in Supplementary Tables 2 and 3, respectively. The sections were viewed and studied under Microscope Slide Scanner (Axioscan 7, ZEISS, Germany) and relative software (ZEISS ZEN, blue edition).

#### Immunohistochemistry staining

CD31, an endothelial cell marker, was staining using immunohistochemistry to study vessel formation. The paraffin Sects. (5 μm) were deparaffinized and filled with citric acid (pH 6.0) antigen retrieval buffer for antigen retrieval in a microwave oven. After cooling to room temperature, the sections were washed three times in PBS (pH 7.4) for 5 min each. To block endogenous peroxidase activity, sections were placed at 3% hydrogen peroxide and incubated at room temperature in darkness for 25 min, then blocked with 5% serum for 30 min. The primary antibody against CD31 rabbit pAb (1:1000, Servicebio, Wuhan Servicebio Technology Co., Ltd.) was added to the sections, and the sections were incubated overnight at 4 ℃. T The sections were coated with goat anti-Rabbit IgG secondary antibody H&L (1:200, Servicebio, Wuhan Servicebio Technology Co., Ltd.) and incubated at 37 °C for 50 min. The sections were developed with 3,3′-Diaminobenzidine (DAB) Tetrahydrochloride Chromogenic Kit (Servicebio, Wuhan Servicebio Technology Co., Ltd.), and the positive is brownish yellow. Finally, sections were stained with hematoxylin and observed under an optical microscope (CX33, Olympus, Japan).

#### Toxicity in vivo

At predetermined postoperative times (days 7, 14, and 21), the rats were sacrificed. The major organs (heart, liver, spleen, lung, kidney, thymus, testis and epididymis) of rats in rcBCS group was dissected and histologically examined for safety in vivo at the end of experiments.

### Animal ethics

Animal experiments were conducted according to the Guidelines for Animal Care and Use Committee of Xiamen University. All animal experiments described were approved by the Animal Experimentation Ethics Committee of Xiamen University, China (XMULAC20190008). The in-vivo study is reported in accordance with the ARRIVE guidelines (Animal Research: Reporting of In Vivo Experiments) for reporting experiments^26,27^.

### Statistical analysis

Statistical analyses were performed with IBM SPSS Statistics for Windows version 24.0 (IBM Corp., USA). In general, the central tendency and discrete state of continuous variables were expressed by mean and standard deviation (SD). The two independent-sample *t*-test and Mann–Whitney U test were used for statistical analysis. Differences were considered significant at p < 0.05. Qualitative examination was used in the rest of the section. All tests were performed at least in triplicate.

## Results

### Preparation of radiation crosslinked bilayer collagen scaffold

The rcBCS was a bilayer membrane system prepared from purified medical grade bovine collagen type I for cutaneous placement and prepared under Good Manufacturing Practice (GMP) requirements and conditions. After preparing rcBCS, it was precisely packaged and irradiated for sterilization.

**Figure 1:**
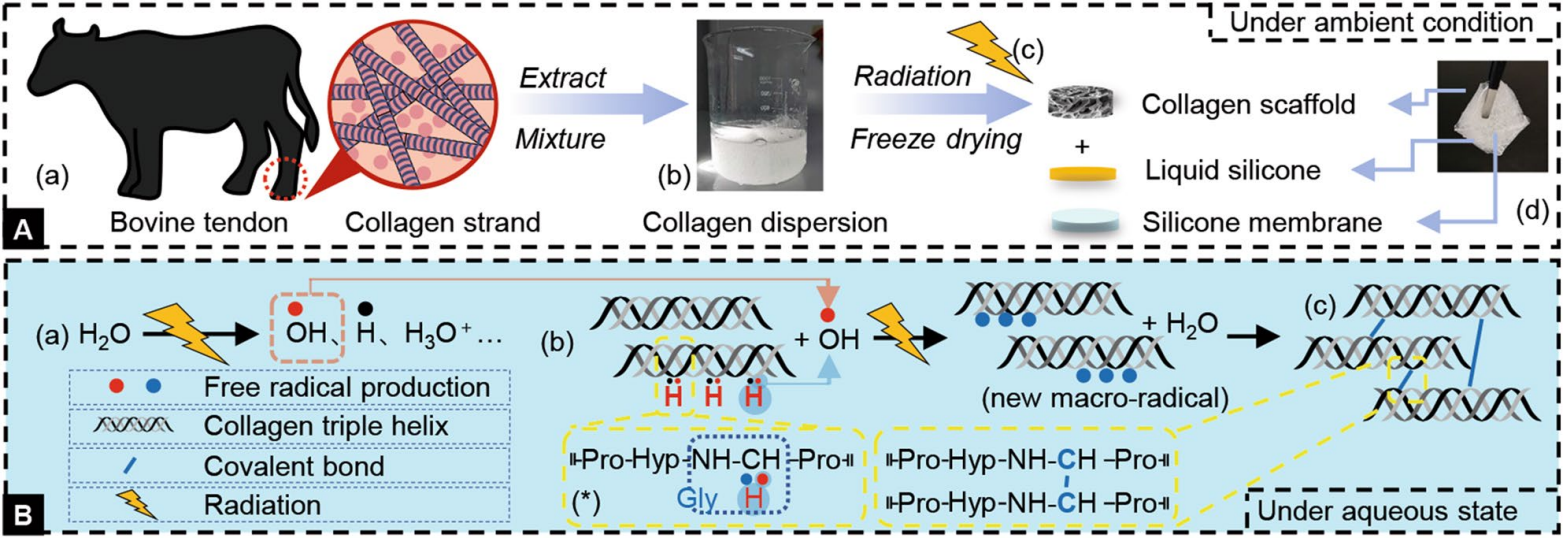
Schematic illustration of the preparation progress of radiation crosslinked bilayer collagen scaffold. (**A**) The preparation process of radiation crosslinked bilayer collagen scaffold. (**a**) Extraction. (**b**) Mixture. (**c**) Radiation. (**d**) Combination. (**B**) The proposed mechanisms of crosslinking for collagen under radiation. (**a**) Water radiolysis. (**b**) Chain-transfer. (**c**) Cross-linking. (*) Representative segment.

Figure 1 described the preparation process of rcBCS, and the mechanisms for crosslinking collagen under radiation. A four-step process was used to prepare rcBCS. First, collagen type I was extracted from bovine tendons. In this study, commercially available collagen was used directly. Next, collagen type I and Milli Q water were mixed to form collagen dispersion, and stirred overnight at 4℃. Then, the collagen dispersion was injected into the molds. The collagen dispersion with mold was irradiated by electron beam to form collagen hydrogel follow with freeze-drying to obtain collagen scaffold. Finally, liquid silicone combined silicone membrane with collagen scaffold to form rcBCS. At the optimal exposure dose (5 k Gy) determined by previous studies, the energy of the electron beam was mostly absorbed by water molecules^19,28^. And water radiolysis generated reactive species such as hydroxyl radical, which interacted with the polypeptide chain^19,29^. The glycine was partially involved in crosslinking, and new macroradicals recombined to form a covalent bond between the polypeptide chains. But the backbone structure of collagen was still maintained^19,28,30,31^.

### Physico-chemical characterization of radiation crosslinked bilayer collagen scaffold

#### Morphology Analysis

**Figure 2:**
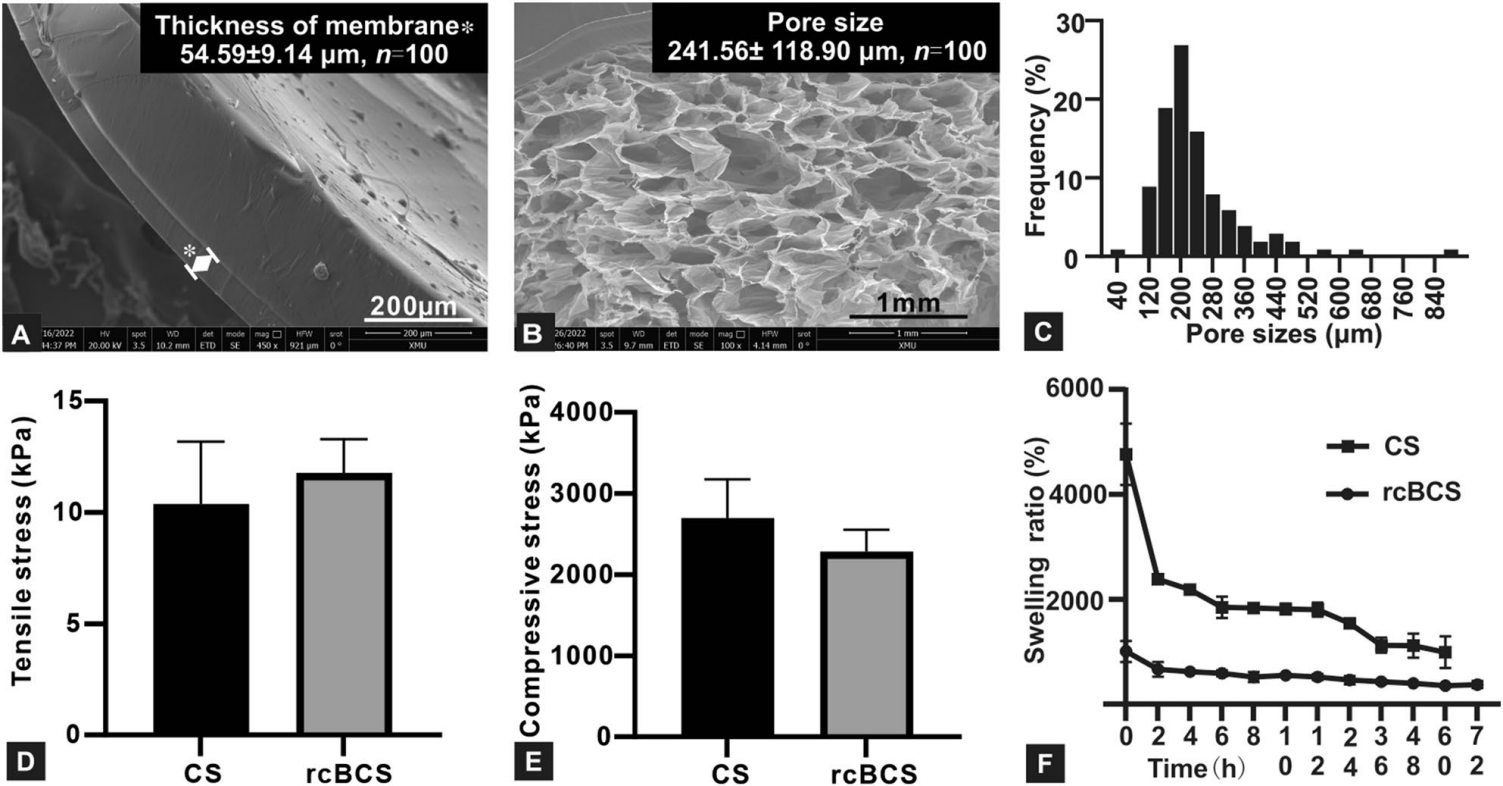
Physico-chemical characterization of radiation crosslinked bilayer collagen scaffold (rcBCS). (**A**) Scanning electron microscopy observation of the microstructures of silicone membrane (*) with liquid silicone. (**B**) Scanning electron microscopy observation of cross-section structure of rcBCS. (**C**) The distribution of pore sizes of rcBCS. (**D**) Tensile stress at maximum load of rcBCS relative to collagen scaffold (CS). (**E**) Compressive stress at maximum load of rcBCS relative to CS. Data are expressed as mean ± SD. (**F**) Swelling ratio of the rcBCS and CS shows high swelling capacity.

The FE-ESEM results revealed that RCBCS cross-sectional profiles had a suitable silicon film thickness and a high porosity microstructure with visibly connected pores (Fig. 2). Surface profiles of collagen scaffold and silicone membrane in rcBCS were shown in Supplementary Figs. 2 and 3, respectively. In general, having a microstructure similar to the host environment means that vascularization begins well and new blood vessels can grow. Notably, the porosity microstructure in rcBCS was morphological and architecturally similar to the natural ECM^32^. And rcBCS further provided a high surface-area -volume ratio in interconnected porous microstructure for cell adhesion and proliferation, such as fibroblasts and vascular endothelial cell. Adequate pore interconnectivity in rcBCS ensured that cells were within 200 µm of the new blood supply for mass transfer of oxygen, nutrients, metabolites, and cellular signals (among other regulatory factors)^32^. In addition, the mean diameter of pore sizes in rcBCS was 241.56 ± 118.90 μm, and the pore distribution was homogeneous. The pore size is suitable, and is between Pelnac® (216.5 ± 30.5 μm, Gunze Medical Materials Center, Kyoto, Japan) and Integra® (133.2 ± 16.8 μm, Integra® Life Sciences Corp., Plainsboro, NJ, USA)^33^. This means that the pores are not too small to occur pore occlusion by the cells. In addition, mechanical properties were tested to ensure that the pore size was not too large to affect the stability of the scaffold.

**Figure 3:**
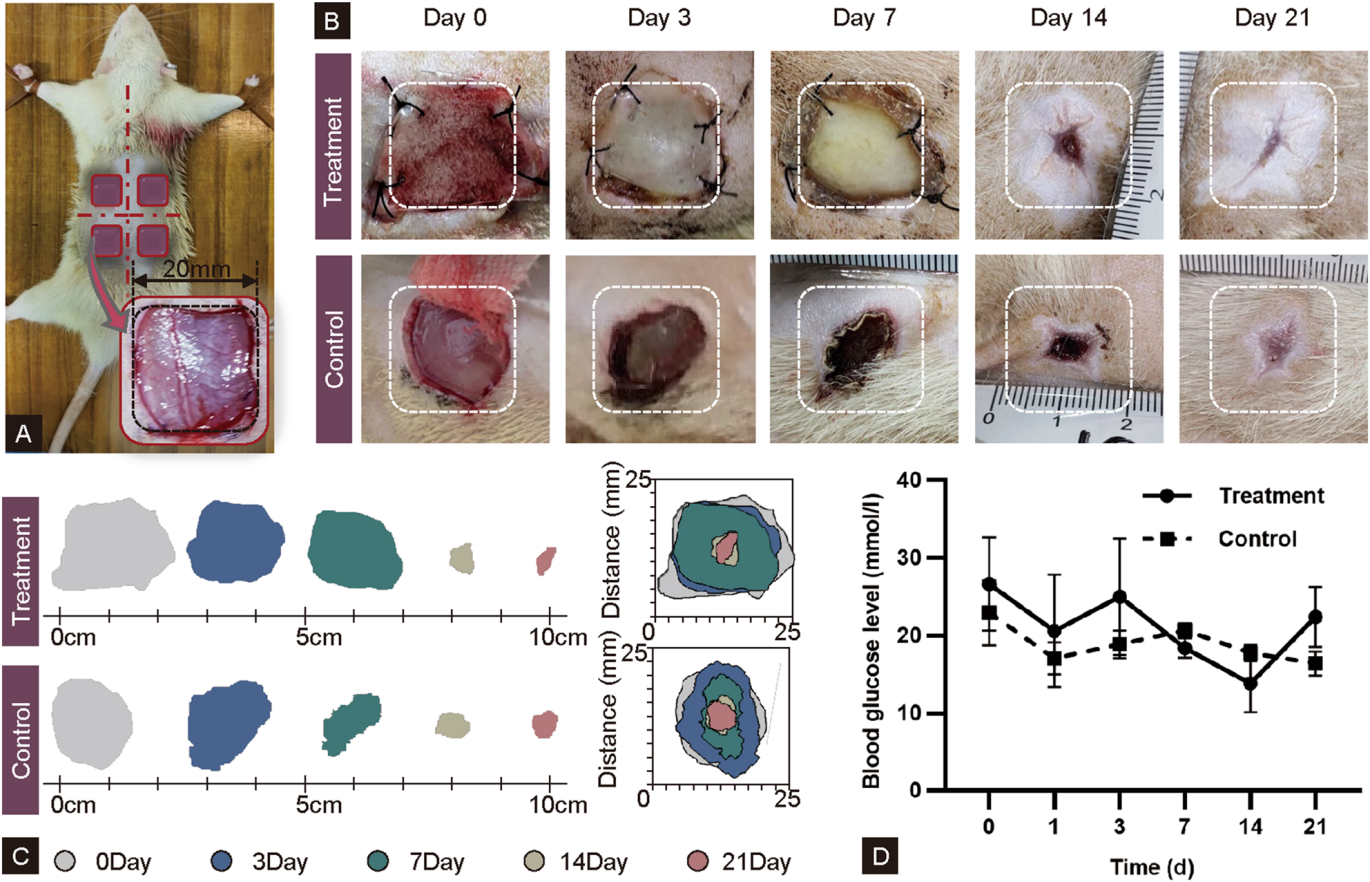
Wound healing in vivo in STZ-induced diabetic rat. (**A**) Schematic illustration of 20 mm diameter and full-thickness wounds created at either side of the dorsal central line. (**B**) Representative photographs of visual appearance of wound excised on rat on days 0, 3, 7, 14 and 21; (**C**) Representative traces of wound-bed closure during 21 days. (**D**) Monitoring of blood glucose level at varied time points. (Treatment = rcBCS group, Control = sham operation group).

#### Mechanical property

The mechanical property of diabetic skin was impaired compared to nondiabetic skin, due to abnormal deposition of ECM and lower levels of collagen^34^. Promoting simultaneous tissue growth, collagen-based scaffolds provided a fundamental mechanism to promote regeneration and stimulate cells^35^. However, a highly porous structure may interfere with the mechanical properties of the scaffold. And the pulling force of cells may cause shape deformation of scaffold with poor mechanical properties. Therefore, mechanical properties were tested to verify that rcBCS had a highly porous structure without sacrificing mechanical properties (Supplementary Fig. 4). The results showed that rcBCS and collagen scaffold had similar tensile stress and compressive stress (Fig. 2) (P > 0.05, Supplementary Table 4), confirming that the application of silicone membrane did not affect the mechanical properties of scaffold. More data, including strain at break and energy at maximum load, can be found in Supplementary Tables 5 and 6. The application of naturally derived collagen showed poor mechanical properties.

#### Swelling ratio

The three-dimensional pore structure of collagen scaffold determined water retention. Immediately after taking in the liquid, collagen scaffold absorbed nearly 47 times (4764.91 ± 477.86%) its dry weight water, reaching equilibrium for 2 h (Fig. 2). It was consistent with the hemostatic needs of the healing process. Because rcBCS contains a silicone membrane without water absorption, rcBCS showed low swelling ratios compared to collagen scaffold. The swelling ratio of collagen scaffold and rcBCS gradually decreased, which may be caused by natural degradation. The results inferred that secondary injury was avoided without removing the scaffold material, and created more room for vascular reconstruction with scaffold degradation. The collagen scaffold structure collapsed after 72 h, while the rcBCS with silicone membrane was still intact. This suggested that the silicone membrane had the potential to play a supporting role and prevent bacteria from infiltrating. Consistent swelling also ensures the benefit of the ability to absorb wound exudate and keep the wound moist, which is beneficial for wound healing^36^.

### Wound modeling evaluation in diabetic cutaneous wound model

#### Diabetic wound healing in vivo

The modeling effectiveness of rcBCS in vivo was examined and summarized on days 0, 7, 14, and 21 to further establish the potential for diabetic wound healing (Supplementary Fig. 5). Creating a model of a complete skin defect in diabetic rats closely parallel the human wound healing process. In sham operation group, Tegaderm™ Film (3M, USA) acted as the outer dressing. A large, full-thick skin defect wound, however, could result in death and other risks to experimental animals in the sham operation group without rcBCS. Thus, the wound sizes in sham operation group (approximately 1.75 cm × 1.75 cm) were smaller than those in rcBCS group (approximately 2 cm × 2 cm) (Fig. 3)^22,26,37,38^. A significant increase in blood glucose levels (> 16.7 mmol/L) demonstrated the successful formation of diabetic rats as previous studies^14^ .

The rcBCS group and sham operation group showed different healing patterns. As a transitional substitute for ECM and excellent for cell attachment and spread, collagen in rcBCS was returned to the wound to restore mechanical properties. The rcBCS was dynamically modified and used in diabetic wound modeling to support tissue growth and resist wound retraction. Conversely, the sham operation group wound remained in a defective state until the formation of the scab on day 7, and the scab was easily broken. The promising ability of wound modeling in rcBCS may attribute to several key characteristics of pure collagen as previously mentioned. In addition, this result indicated rcBCS had certain positive wound modeling effects with scaffold degradation. Pure composition of collagen and morphological structure could further magnify the wound modeling effects of rcBCS as its proangiogenic activity.

#### Histopathological analysis

**Figure 4:**
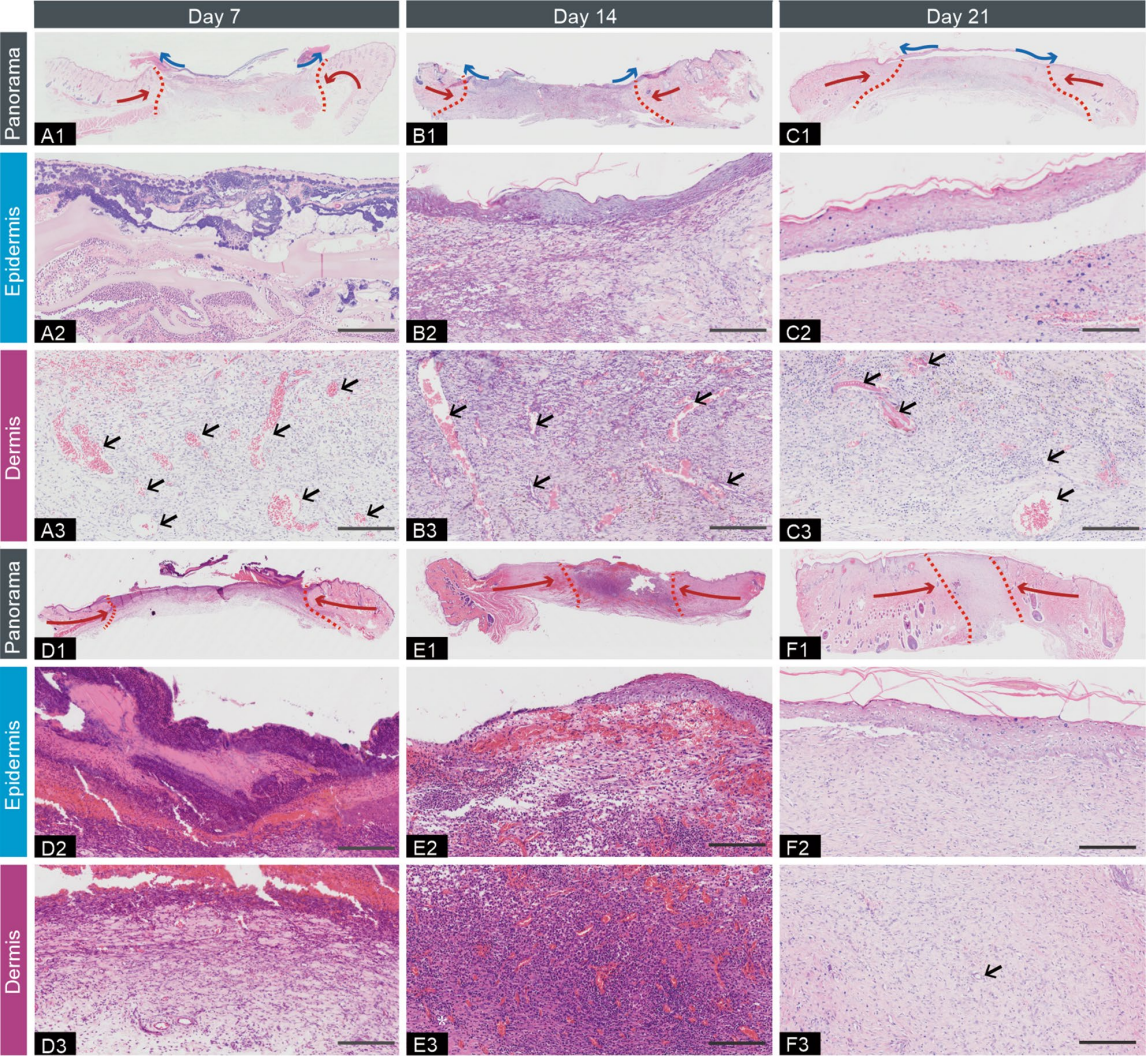
Representative images of H&E staining. (**A**, **B**, **C** represent rcBCS group, and **D**, **E**, **F** represent sham operation group; 1 represents panorama image, 2 represents epidermis image, 3 represent dermis image. Blood vessels: black arrow; Inflammatory cell: *; The blue lines indicate the support force of bilayer dermal equivalent; The red lines indicate the skin contraction force; The areas inside the red dashed lines indicate granulation tissue. Bar = 200 μm).

Physiological wound healing is characterized by sequential, yet overlapping, stages of hemostasis, inflammation, proliferation, and remodeling^39,40^. Histopathological analysis was carried out to further verify the effect of rcBCS on wound healing in diabetes The results of H&E staining were shown in Fig. 4. The thick residual scab and very thin dermal structure were observed in sham operation group on day 7. Scabs in the sham operation group, which contained dried hemocytes, exudates, serum, and acute inflammatory cells, were common to damage and caused erosion. Comparatively, no residual scabs (mainly residual silicone membrane) were observed in rcBCS group. The dermal structure in sham operation group was much thinner than that in rcBCS group. And in sham operation group, a large number of inflammatory cells were accumulated in the granular structure and dermal structure, but few in rcBCS group. The results confirmed that diabetic wound healing is delayed and the rcBCS played a role in dermal repair and transitional ECM template in the early healing stage. This also supported the results of the differences in healing patterns. Meanwhile, mild inflammatory microenvironment and moderate neovascularization were observed in rcBCS group on day 7, while massive inflammatory cells infiltrated dermal structure and few blood vessels were observed in sham operation group. It favored that rcBCS was effective for diabetic wound healing to some extent even in the early stages^17,41–44^. When the residual scab was still in the sham operation group on day 14, a thin layer of corn was form in rcBCS group. As rcBCS degraded, a mild inflammatory microenvironment was displayed and there was more room for blood vessel formation and subcutaneous layer accumulation. In contrast, there are still a large number of inflammatory cell infiltration in sham operation group. And this further confirmed that the process of diabetic wound healing was delayed in the inflammatory phase. During the days 7 through 21, vascularization was high in rcBCS group. On day 21, the damaged full-skin defect was reconstructed. Although cornification was observed, there was no significant vascularization in the control group.

**Figure 5:**
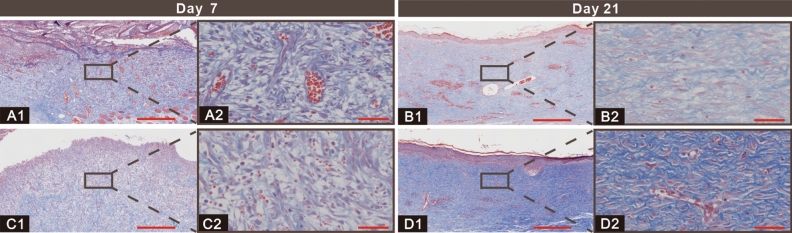
Representative images of the Masson’s trichrome staining on the day 7 and 14 (**A**, **B** represent rcBCS group and **C**, **D** represent sham operation group; Bar in 1 = 500 μm, bar in 2 = 50 μm).

Masson's trichrome stain indicated collagen deposition at the wound sites. Collagen deposition was thought to be an intuitive assessment indicator of wound modeling^6^. Images of Masson's trichrome staining showed that collagen deposition was accelerated by rcBCS compared to sham operation group (Fig. 5). In cases where healing is compromised, the application of rcBCS showed a greater positive effect in enhancing collagen deposition.

#### Angiogenesis analysis in vivo

**Figure 6:**
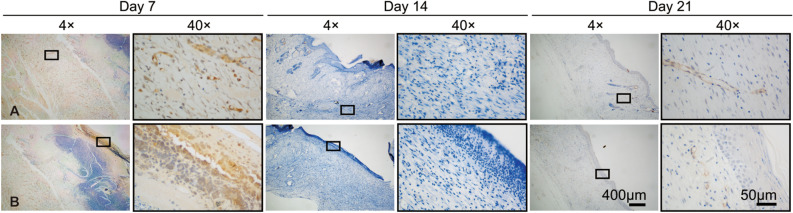
Representative immunohistochemical staining images of CD31 on (**A**) dermis and (**B**) epidermal layer on days 7, 14 and 21 in rcBCS group.

A further angiogenesis test was performed using angiogenesis cytokine expression, such as CD31 A significant role in diabetic wound healing and tissue remodeling is played by angiogenesis, which occurs throughout the wound healing process as blood vessels grow. CD31 is the common indicator for vascular endothelial cells^3^. Consequently, CD31 expression levels were determined by immunohistochemical staining on days 7, 14 and 21 (Fig. 6). A high-level CD31 expression was displayed in rcBCS group on day 7. A similar result to higher revascularization in rcBCS group was observed by H&E staining. In rcBCS group, CD31 expression decreased gradually with material degradation (day 14). At this point, the new blood vessels, according to H&E staining results, were already sufficient to accommodate the need for many cells within 200 µm of the blood for the transfer of oxygen, nutrients, waste, and carbon dioxide. CD31 expression levels increased on day 21. The encouraging findings were consistent with other studies^1,3,6^.

#### Safety evaluation by toxicity testing in vivo

Histological examination of rat organs (heart, liver, spleen, lung, kidney, thymus, testis and epididymis) was performed, and the results further indicated negligible in vivo toxicity of rcBCS implantation (Supplementary Figs. 6 to 9). Besides, the cytotoxic effect of the rcBCS in vitro cell culture was investigated in a previous study, which showed good cellular compatibility^20^.

## Discussion

**Figure 7:**
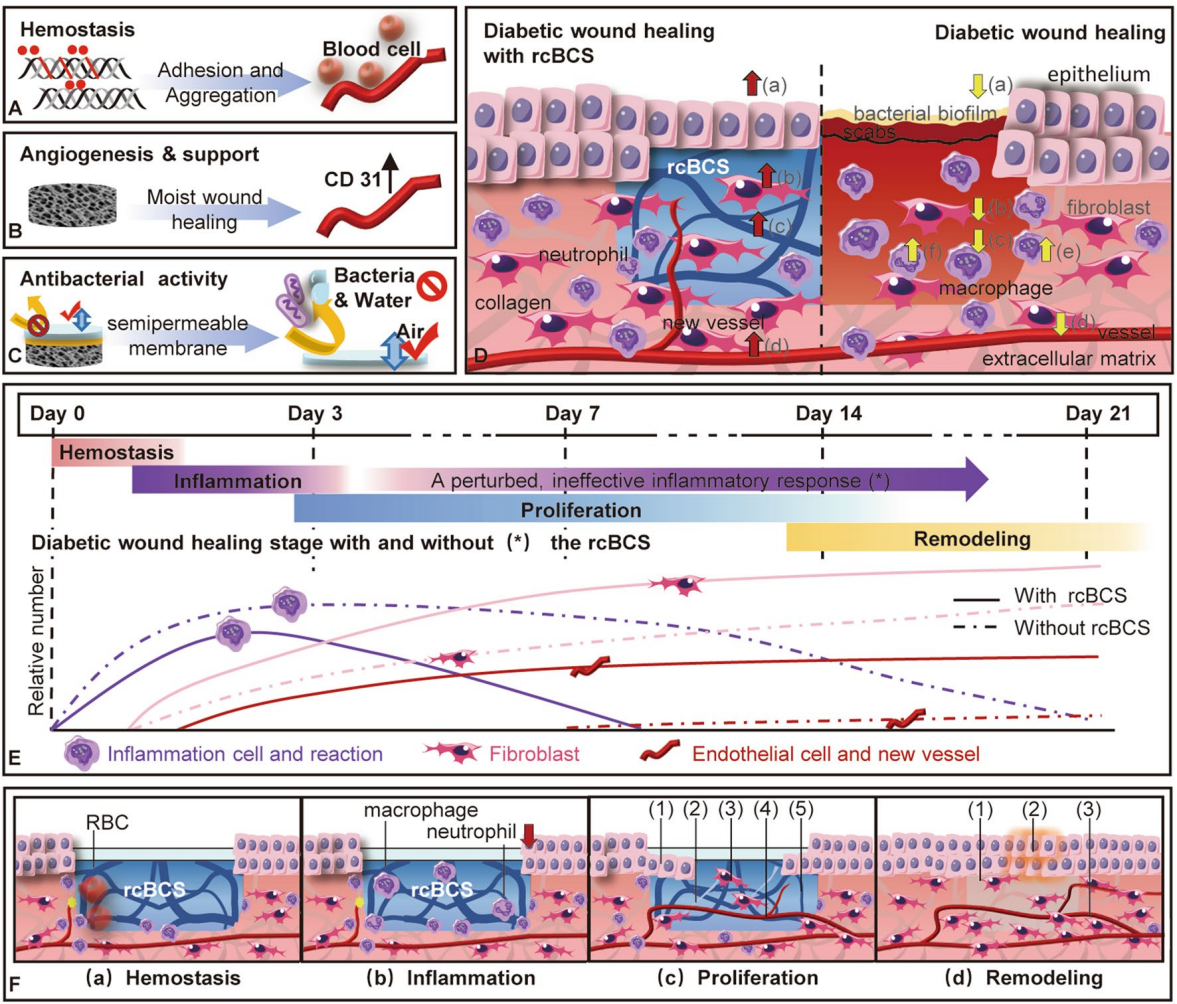
The possible cellular mechanisms involved in diabetic wound healing with treatment of rcBCS. (**A**) Hemostasis ability due to the high swelling ratio. (**B**) Facilitating vascularization and providing mechanical support. (**C**) Antibacterial ability due to silicone membrane. (**D**) In comparison with impaired healing in diabetic wound (right), the healing ability is improved for the treatment of rcBCS (left). (**a**) Keratinocyte migration. (**b**) Fibroblast proliferation and attachment. (**c**) Collagen deposition. (**d**) Endothelial cell migration. (**e**) Inflammation. (**f**) Neutrophil removed. (**E**) The temporal sequence of overlapping processes involved in the healing of diabetes wound with and without the treatment of rcBCS. (**F**) The interaction of rcBCS with cells during diabetes healing processes. (**a**) Hemostasis. (**b**) Inflammation. (**c**) Proliferation. (1) advancing epithelial layer. (2) collagen deposition. (3) increased fibroblast activity. (4) new vessel. (5) degradation of rcBCS. (**d**) Remodeling. (1) reconstruction of the ECM. (2) superficial scar. (3) rich in blood vessels.

Overall, rcBCS demonstrated a good level of vascularization and healing. This suggests that collagen matrix plays a pivotal role in stimulating angiogenic processes. The initial transition from inflammatory to proliferative phase of wounds was mediated by the pure collagen component, perfectly mimicking the structure of ECM, even when the wound was in the inflammatory phase (day1 through day3). Particularly evident was the rapid development of surrounding normal tissue towards the wound center with rcBCS degradation and epithelium growth. ECM was continuously secreted and proliferated by fibroblasts in order to reconstruct the normal dermal structure. Therapy with rcBCS was beneficial for dermal reconstruction and fibroblasts migration. In addition, the rapidly high swelling ratio of rcBCS can assist in early hemostasis. Bacteria and water are prevented by the dense silicone membrane, which also ensures the exchange of air. The dense silicone membrane prevents bacteria and water, while ensuring the exchange of air (Fig. 7). Despite positive impact on wound remodeling, future research also needs to investigate the underlying mechanism with the application of rcBCS.

## Conclusions

No cells or viable biomolecules were present in rcBCS. Collagen in rcBCS is crosslinked with radiation, imparting strength and stability. In summary, the results obtained in this study provide evidence that rcBCS promotes ECM remodeling. As a result of these characteristics, rcBCS may be beneficial for rebalancing the chronic wound microenvironment and promoting vascularization, particularly in chronic diabetic wounds. Tissue development is accelerated by angiogenesis, which allows the transport of nutrients and metabolic waste. It has great potential for surgeons in the future to use the novel rcBCS to treat diabetic wounds with impaired angiogenesis. Also, the preparation process is less expensive, more efficient, and greener. There is considerable therapeutic potential for treating diabetic wounds with rcBCS.

## Supplementary Information

Supplementary Information.

## Data Availability

The data presented in this study are available on request from the corresponding author.

## Abbreviations

ECM: Extracellular matrix
FDA: Food and Drug Administration
Gly: Glycine
GMP: Good manufacturing practices
H&E: Hematoxylin and eosin
Hyp: Hydroxyproline
IL-6: Interleukin 6
Pro: Proline
rcBCS: Radiation crosslinked bilayer collagen scaffold
SD: Sprague Dawley
STZ: Streptozotocin
TNF-α: Tumor necrosis factor alpha
