# Supplementary material for: Qualitative study on diabetic cutaneous wound healing with radiation crosslinked bilayer collagen scaffold in rat model
Source: Sci Rep. 2023 Apr 19;13:6399. doi: 10.1038/s41598-023-33372-z (PMC10115801; doi:10.1038/s41598-023-33372-z)
Supplement: Supplementary file 1 — Supplementary Information. [file 41598_2023_33372_MOESM1_ESM.docx]

**Supplementary**

A qualitative study on diabetic cutaneous wound healing with radiation crosslinked bilayer dermal/collagen scaffold: implications for collagen-based scaffold application

Hongwei Li ^1^, Xin Chen ^2^, Kang Ren ^1^, Lihao Wu ^1^, Gong Chen ^1^, Ling Xu ^1,3^* (*corresponding author)

^1^ State Key Laboratory of Molecular Vaccinology and Molecular Diagnostics, Department of Laboratory Medicine, School of Public Heath, Xiamen University, Xiamen, 361102, P. R. China.

2 Department of Burn, Beijing Jishuitan Hospital, Beijing 100035, P. R. China.

3 Shenzhen Research Institute of Xiamen University, Shenzhen 51800, P. R. China.

**SUPPLEMENTARY - CONTENTS**

| 1 | **Abbreviations** | Page 1 |
| --- | --- | --- |
| 2 | **Packaging and sterilization of rcBCS** | Page 2 |
| 3 | **Randomization and groups** | Page 4 |
| 4 | **Hematoxylin-eosin and Masson's trichrome staining procedures** | Page 5 |
| 5 | **Microstructure of surface profiles by scanning electron microscopy** | Page 7 |
| 6 | **Mechanical property** | Page 9 |
| 7 | **Wound area measurement** | Page 12 |
| 8 | **Safety evaluation by cytotoxicity testing *in vitro* and toxicity testing *in vivo*** | Page 13 |

# 1. Abbreviations

ECM = Extracellular Matrix;

FDA = Food and Drug Administration;

Gly = Glycine;

GMP = Good Manufacturing Practices;

H&E = Hematoxylin and Eosin;

Hyp = Hydroxyproline;

IL-6 = Interleukin 6;

Pro = Proline;

rcBCS = radiation crosslinked bilayer dermal/collagen scaffold;

SD = Sprague Dawley;

STZ = Streptozotocin;

TNF-α = Tumor Necrosis Factor Alpha.

# 2. Packaging and sterilization of rcBCS

In order to supply rcBCS to different areas without biobanks or storage facilitiest, no cell or viable biological molecules were present within rcBCS. In addition, achieving rcBCS was broadly available, compatible packaging and sterilization processes was developed.

After manufacture, rcBCS is stored in a sterilized, de-pyrogen and sealed blister packed container with Dupont Tyvek paper (Supplementary Fig. 1. A). Indicator of radiation sterilization is affixed to the blister packed container (Supplementary Fig. 1. B). The entire package is inserted into a tamper-evident foil pouch for protection during transportation and storage. And instructions for use is attached to the outside of foil pouch (Supplementary Fig. 1. C).


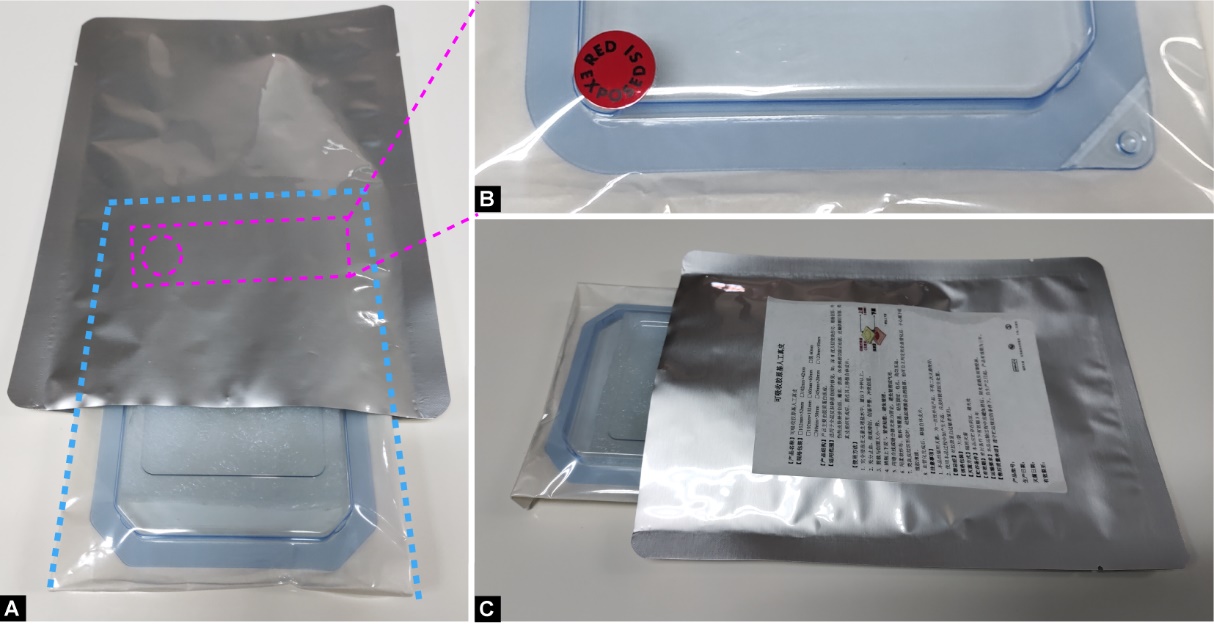


**Supplementary Figure 1**. Packaging of the sealed and sterilized rcBCS for storage or shipping prior to use. (A) Medical-grade blister pack containing rcBCS (unsealing for visualization purposes only; for medical use, the entire package is sealed and verified according to ISO/TS 16775:2021and ISO 11607-1:2019). (B) That indicator of irradiation sterilization turns red represents the sterilization dose is reached. (C) Labels on the back of the outer pack with specifications, expiry date, direction for use and traceable ID.

To provide an additional level of medical device safety beyond an aseptic manufacturing process, end-stage sterilization procedure is applied . Electron beam irradiation has widely used for medical device sterilization and is gaining popularity as a sterilization method. And previous experiments have shown that electron beam irradiation is suited for soft, tissue-engineered devices at a validated range of irradiation dose. Sterilization validation of rcBCS was performed according to ISO 14937:11 (R2016) and ISO 11137-1-3:2017 sterilization standards.

Quality control of samples and the packaging was conducted by visual inspection, mechanical, swelling tests on the electron beam irradiation exposed samples and non-sterilized samples as controls. The sterilization doses of samples were strictly detected by radiochromic film dosimetry (FTR-125 Fuji CTA Film, Fuji Film Corporation, Japan). Additionally, indicator of radiation sterilization was taken to verify the sterilization quality, and was affixed to the blister packed container (Supplementary Fig. 1. B).

# 3. Randomization and groups

Diabetic rats were designated randomly as either rcBCS treatment group or sham operation control group by coin toss. Previous studies served as a reference in determining sample size ^1,2^. In each group, there were at least twelve study sites available at any given time (Supplementary Tab.1). About 21 days are required for complete healing, according to preliminary findings. The maximum observation time was 21 days.

**Supplementary Table 1.**Experimental group and schedule

| **Schedule ^a^** | | **Group** | | |
| --- | --- | --- | --- | --- |
|  |  | **rcBCS treatment group** |  | **sham operation control group** |
| Day 0 | *N*_1_ | 9 |  | 9 |
|  | *N*_2_ | 36 |  | 36 |
|  | *n*_1_ | 0 |  | 0 |
|  | *n*_2_ | 0 |  | 0 |
| Day 3 | *N*_1_ | 9 |  | 9 |
|  | *N*_2_ | 36 |  | 36 |
|  | *n*_1_ | 0 |  | 0 |
|  | *n*_2_ | 0 |  | 0 |
| Day 7 | *N*_1_ | 9 |  | 9 |
|  | *N*_2_ | 36 |  | 36 |
|  | *n*_1_ | 3 |  | 3 |
|  | *n*_2_ | 12 |  | 12 |
| Day 14 | *N*_1_ | 6 |  | 6 |
|  | *N*_2_ | 24 |  | 24 |
|  | *n*_1_ | 3 |  | 3 |
|  | *n*_2_ | 12 |  | 12 |
| Day 21 | *N*_1_ | 3 |  | 3 |
|  | *N*_2_ | 12 |  | 12 |
|  | *n*_1_ | 3 |  | 3 |
|  | *n*_2_ | 12 |  | 12 |

a. *N*_1_= Total number of laboratory animals; *N*_2_= Total number of wounds (especially for postoperative general observation); *n*_1_= Sacrificial number of laboratory animals; *n*_2_= Number of wounds harvested after animal sacrifice (especially for biological sample acquisition).

# 4. Hematoxylin-eosin and Masson's trichrome staining procedures

Sections were stained with hematoxylin-eosin (Supplementary Tab.2) and Masson’s trichrome staining (Supplementary Tab.3) according to research experience and previous reports.

**Supplementary Table 2**. Procedure of hematoxylin-eosin staining

| **Step** | **Description** |
| --- | --- |
| 1 | Xylene I (Sinopharm Chemical Reagent Co. Ltd., China) for 20 min;  Xylene II (Sinopharm Chemical Reagent Co. Ltd., China) for 20 min;  100% ethanol I (Sinopharm Chemical Reagent Co. Ltd., China) for 5 min;  100% ethanol II (Sinopharm Chemical Reagent Co. Ltd., China) for 5 min;  75% ethanol (Sinopharm Chemical Reagent Co. Ltd., China) for 5 min;  Rinsing with tap water. |
| 2 | After 3-5 minutes of staining with Hematoxylin solution(Servicebio, Wuhan Servicebio Technology Co., Ltd.), rinse with tap water.  Then treat the section with Hematoxylin Differentiation solution (Servicebio, Wuhan Servicebio Technology Co., Ltd.), rinse with tap water.  Treat the section with Hematoxylin Scott Tap Bluing(Servicebio, Wuhan Servicebio Technology Co., Ltd.), rinse with tap water. |
| 3 | 85% ethanol (Sinopharm Chemical Reagent Co. Ltd., China) for 5 min;  95% ethanol (Sinopharm Chemical Reagent Co. Ltd., China) for 5 min;  Stain with Eosin dye (Servicebio, Wuhan Servicebio Technology Co., Ltd.) for 5 min. |
| 4 | 100% ethanol I (Sinopharm Chemical Reagent Co. Ltd., China) for 5 min;  100% ethanol II (Sinopharm Chemical Reagent Co. Ltd., China) for 5 min;  100% ethanol III (Sinopharm Chemical Reagent Co. Ltd., China) for 5 min;  Xylene I (Sinopharm Chemical Reagent Co. Ltd., China) for 5 min;  Xylene II (Sinopharm Chemical Reagent Co. Ltd., China) for 5 min;  Finally seal with neutral gum (Sinopharm Chemical Reagent Co. Ltd., China). |
| 5 | Under the microscope, nucleus are stained blue, while cytoplasm are stained red. |

**Supplementary Table 3**. Procedure of Masson’s trichrome staining

| **Step** | **Description** |
| --- | --- |
| 1 | Xylene I (Sinopharm Chemical Reagent Co. Ltd., China) for 20 min;  Xylene II (Sinopharm Chemical Reagent Co. Ltd., China) for 20 min;  100% ethanol I (Sinopharm Chemical Reagent Co. Ltd., China) for 5 min;  100% ethanol II (Sinopharm Chemical Reagent Co. Ltd., China) for 5 min;  75% ethanol (Sinopharm Chemical Reagent Co. Ltd., China) for 5 min;  Rinsing with tap water. |
| 2 | Overnight, soak the slices in Masson A (Servicebio, Wuhan Servicebio Technology Co., Ltd.) and rinse them with tap water. |
| 3 | According to a 1:1 ratio, Masson B (Servicebio, Wuhan Servicebio Technology Co., Ltd.) and Masson C (Servicebio, Wuhan Servicebio Technology Co., Ltd.) were created into Masson solution. Then, stain with Masson solution for 1 min, rinse with tap water. Separate it with alcohol containing 1% hydrochloric acid and rinse it with tap water. |
| 4 | In Masson D (Servicebio, Wuhan Servicebio Technology Co., Ltd.), soak the slices for 6 minutes, rinse them with tap water, use Masson E (Servicebio, Wuhan Servicebio Technology Co., Ltd.) for 1 minute, and use Masson F (Servicebio, Wuhan Servicebio Technology Co., Ltd.) for 2–30 seconds. |
| 5 | 1% glacial acetic acid (Servicebio, Wuhan Servicebio Technology Co., Ltd.) was used to rinse the slices before two cups of anhydrous ethanol were used to dehydrate them. |
| 6 | Seal with neutral gum (Sinopharm Chemical Reagent Co. Ltd., China)after 5 minutes of 100% ethanol (Sinopharm Chemical Reagent Co. Ltd., China), 5 minutes of Xylene (Sinopharm Chemical Reagent Co. Ltd., China). |
| 7 | Under the microscope, collagen, fibers, and muscle are stained blue, while fibers, cellulose, and erythrocyte are stained red. |

# 5. Microstructure of surface profiles by scanning electron microscopy

Surface profiles of collagen scaffold in rcBCS obtained by scanning electron microscopy (Supplementary Fig. 2) revealed collagen fibrils after freeze drying with approximately 50-100 nm diameter on surface of rcBCS, while fibrils within the bulk had about 100 nm diameter ^3^.


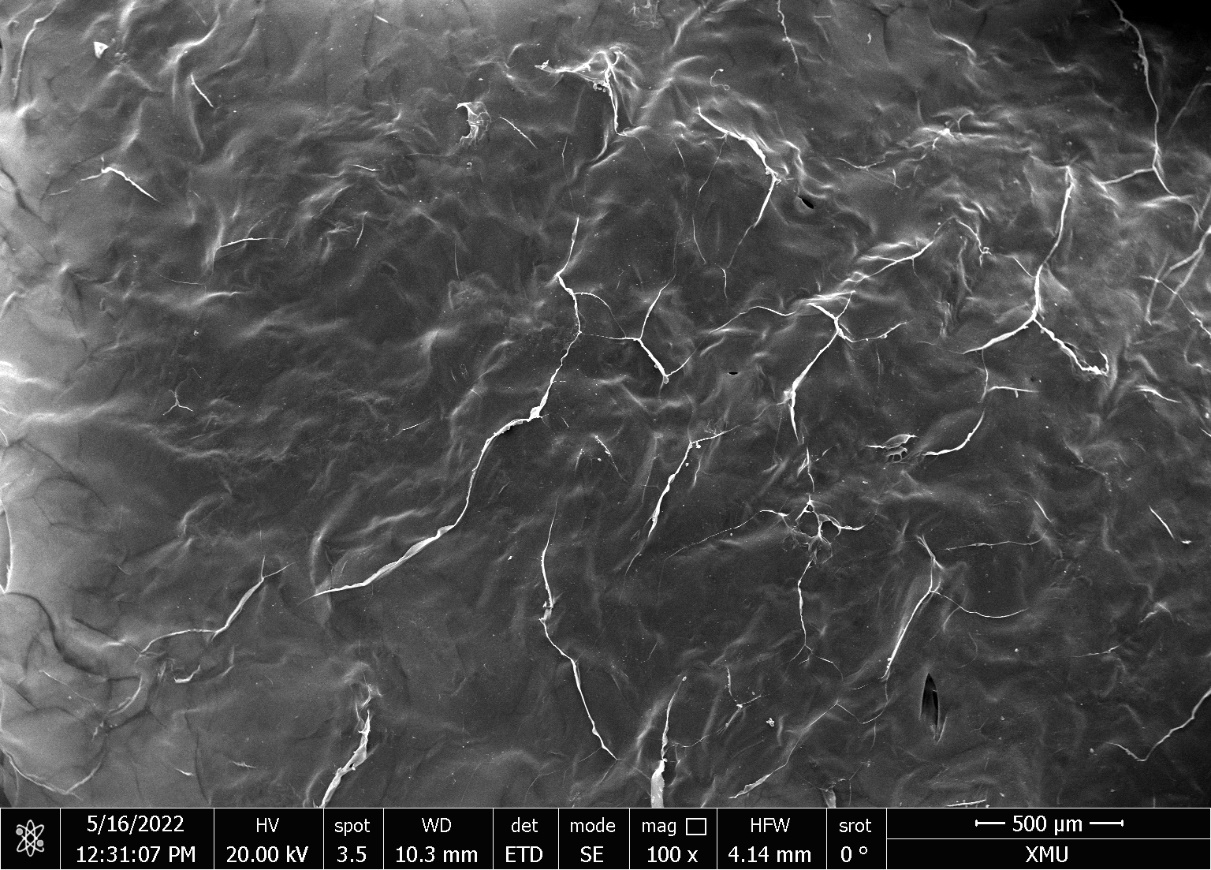


**Supplementary Figure 2**. Scanning electron microscope images of the surface structure of collagen scaffold in rcBCS.

Surface profiles of silicone membrane in rcBCS obtained by scanning electron microscopy (Supplementary Fig. 3) revealed no obvious pores larger than the average size of most bacteria (0.5 - 5μm, diameter) on surface of rcBCS. No obvious pores larger than the average size of most bacteria (0.5 - 5μm, diameter) on the surface of silicone membrane. Due to the dense and non-porous structure, the soft silicone membrane functions to control moisture loss and protect against bacteria and dirt. In addition, it can physically block common gram-negative pathogenic bacteria which cause clinical diabetic ulcer infection, such as staphylococcus aureus (0.8μm in diameter), E. coli bacteria (1.1 -1.5μm in diameters) and pseudomonas aeruginosa (1.5 - 5.0μm in diameters). The average thickness of the silicone membrane is 54μm, and the thickness of silicone membrane and liquid silicone is close to the size of the normal human epidermis (100 – 300μm).


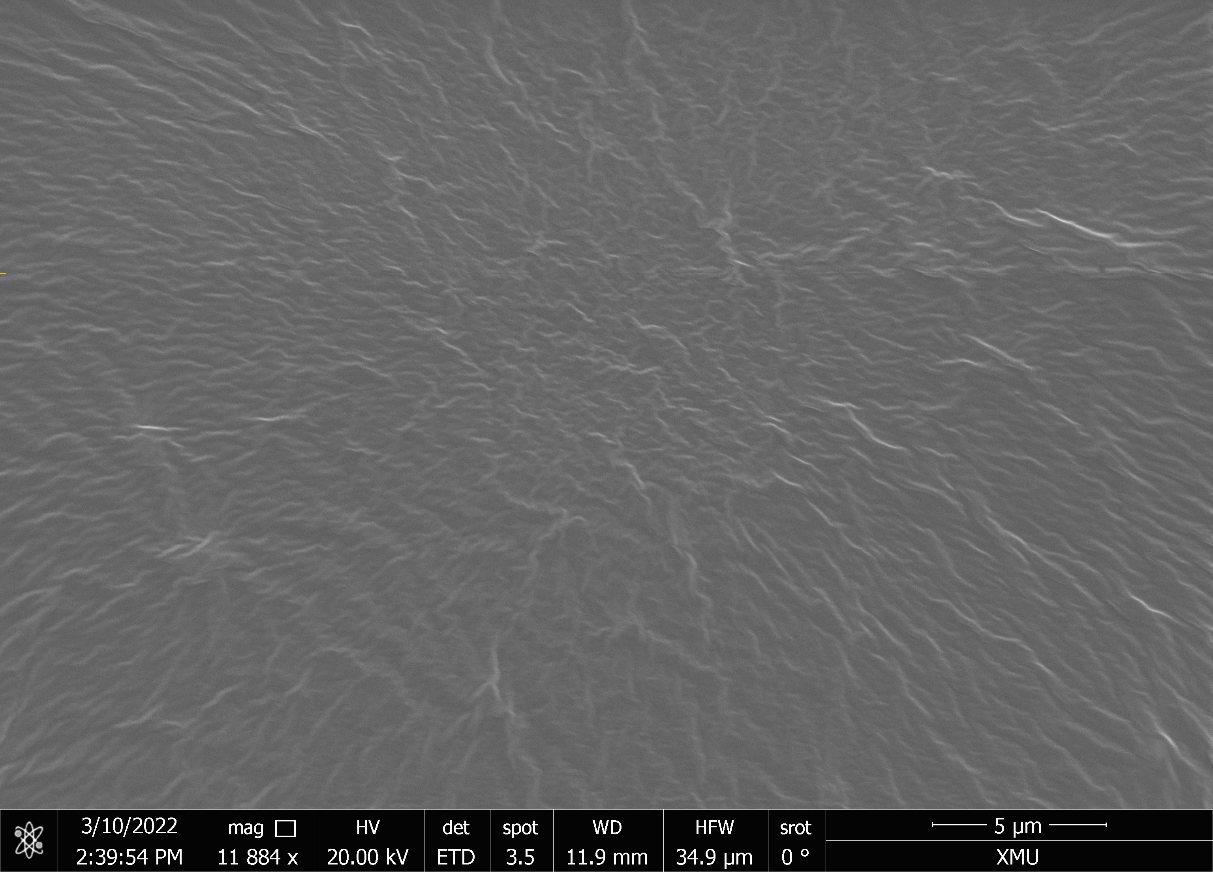


**Supplementary Figure 3**. Scanning electron microscope images of the outside surface structure of silicone membrane in rcBCS.

# 6. Mechanical property

The compression and tension test were carried out by a TA.XTplus100C texture analyzer (Stable Micro Systems, Godalming, UK) for texture evaluation of collagen scaffold and rcBCS. (Supplementary Figure 4)


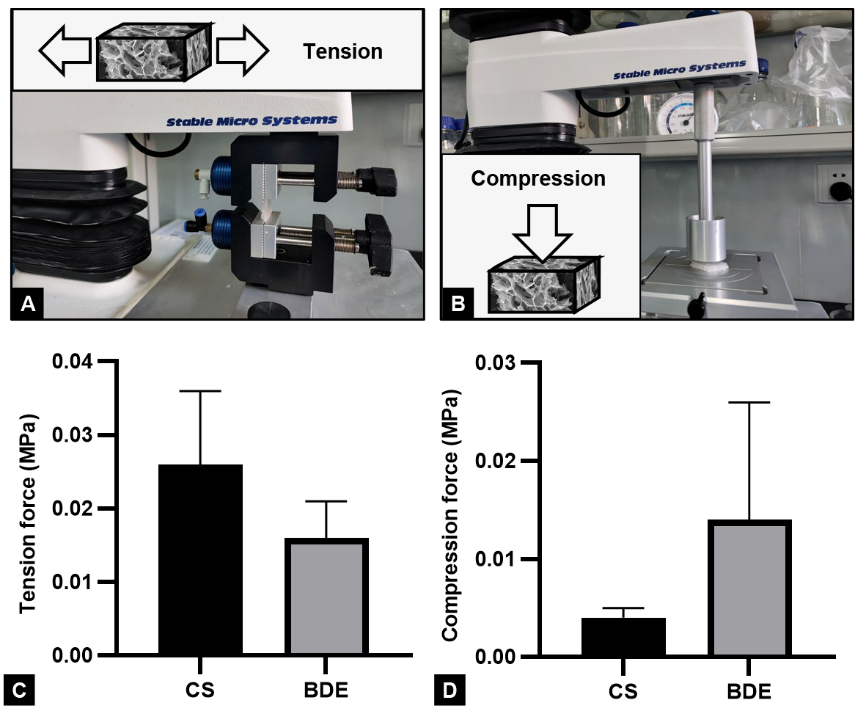


**Supplementary Figure 4**. Illustration of the two methods used to measure the anisotropy of the Young's modulus : TA.XTplus100C texture analyzer measuring force at (A) tension and (B) compression. (Xiamen University)

The silicone membrane was added upon the collagen scaffold, making the structure of rcBCS more biologically similar to human skin, without reducing mechanical properties (*P*>0.05; Raw data and statistical method were shown at Supplementary Tab. 4).

**Supplementary Table 4**. Comparison of the properties of rcBCS and collagen scaffolds

|  | Tensile Stress at Maximum Load (kPa) ^a^ | |  | Compressive Stress at Maximum Load (kPa) ^b^ | |
| --- | --- | --- | --- | --- | --- |
| Group | Collagen scaffold | rcBCS |  | Collagen scaffold | rcBCS |
| raw data ^c^ | 6.26 | 12.36 |  | 2795.74 | 1955.02 |
|  | 9.39 | 10.21 |  | 1857.24 | 2218.08 |
|  | 11.25 | 12.19 |  | 2935.61 | 2194.40 |
|  | 11.15 | 10.35 |  | 2966.81 | 2376.26 |
|  | 13.85 | 13.81 |  | 2937.42 | 2684.78 |
| statistical ^d^ | two independent-sample t-test | |  | two independent-sample t-test | |
| *P* (*t*) | 0.353 > 0.05 (0.987) | |  | 0.129 > 0.05 (1.691) | |
| statistical ^e^ | Mann-Whitney U test | |  | Mann-Whitney U test | |
| *P* (*Z*) | 0.465> 0.05 (0.731) | |  | 0.117> 0.05 (1.567) | |

a. measurements were performed until the samples were ruptured.

b. measurements were performed at 200% relative deformation.

c. one sample was discarded after measurement, and only one result was obtained. A total of 20 samples were used to obtain the above raw data.

d &e. two different statistics methods were used and the same conclusions were obtained (silicone membrane did not reduce the mechanical properties, *P*>0.05).

The strength, breaking strain and toughness of rcBCS and collagen scaffold can be seen at Supplementary Tab.5 & 6. Under the compressive stress of 2 times its own deformation, it still maintains good resilience and resistance to mechanical damage.

**Supplementary Table 5**. Tensile measurements

|  | Tensile Stress at Maximum Load ^a^  (kPa) | Tensile Strain at Maximum Load ^a^  (%) | Energy at Maximum Load ^a^  (kJ/m^3) |
| --- | --- | --- | --- |
| collagen scaffold | 10.381 ± 2.801 | 31.628 ± 5.304 | 0.5 ± 0.47 |
| rcBCS | 11.785 ± 1.512 | 127.419 ± 72.93 | 11.861 ± 6.963 |

a. measurements were performed until the samples were ruptured.

**Supplementary Table 6**. Compressive measurements

|  | Compressive Stress at Maximum Load ^a^  (kPa) | Compressive Strain at Maximum Load ^a^  (%) | Energy at Maximum Load ^a^  (kJ/m^3) |
| --- | --- | --- | --- |
| collagen scaffold | 2698.565 ± 474.991 | 1431.614 ± 403.471 | 684.586 ± 164.229 |
| rcBCS | 2285.706 ± 269.129 | 1824.443 ± 383.675 | 903.651 ± 135.013 |

a. measurements were performed at 200% relative deformation.

# 7. Wound area measurement

An excision wound margin was tracked after wound creation using Photoshop (Adobe systems incorporated, USA) and area was measured. An algorithm was used to remove variations in baseline (day 0) for each wound size to take them into account.

Wound area closed (%, each wound) _day X_ = Wound area (pixel) _day X_ / Wound area (pixel) _day 0_ × 100%. (Wound area (pixel) _day X_ present the relative number of pixels of the wound on day X, which was measured by Photoshop. Wound area (pixel) _day 0_ present the relative number of pixels of the wound on day 0 (baseline), which was measured by Photoshop. )

Differences between the treatment and control groups were calculated, and the data were expressed as means and standard deviations (Supplementary Fig.5).

There was no significant statistical difference with two independent-sample *t*-test. This shows that Tegaderm ^TM^ Film (3M, USA) has anti-contractility, and the method is feasible.


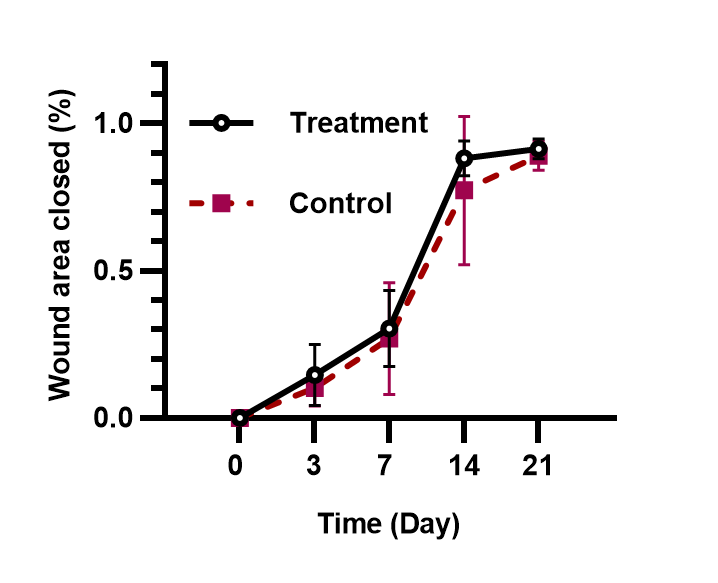


**Supplementary Figure 5**. The rate of wound area closed. (Treatment=rcBCS group; control= sham operation group)

# 8. Safety evaluation by cytotoxicity testing *in vitro* and toxicity testing *in vivo*

According to ISO 10993-5: 2009 (GB/T 16886.5:2017), a panel of third-party certified medical device tests was conducted to quantify the cytotoxicity by MTT assay. The rcBCS exhibited 88% (higher than 80%) of [cell viability](https://www.sciencedirect.com/topics/biochemistry-genetics-and-molecular-biology/cell-viability), demonstrating that the rcBCS was no observable cytotoxicity. Furthermore, the cytotoxic effect of the scaffold *in vitro* cell culture was investigated in in a previous study ^4^.

Histological examination of rat organs (heart, liver, spleen, lung, kidney, thymus, testis and epididymis) was conducted after implanting rcBCS for day 7, 14, 21 (Supplementary Fig.6, Fig7 and Fig.8). The rat organs in the sham operation group at day 21 after surgery are shown in Supplementary Fig.9. No adverse organ damage or inﬂammatory reactions had occurred on days 7, 14, 21 after implanting rcBCS. These results further indicated negligible *in vivo* toxicity of rcBCS implantation.


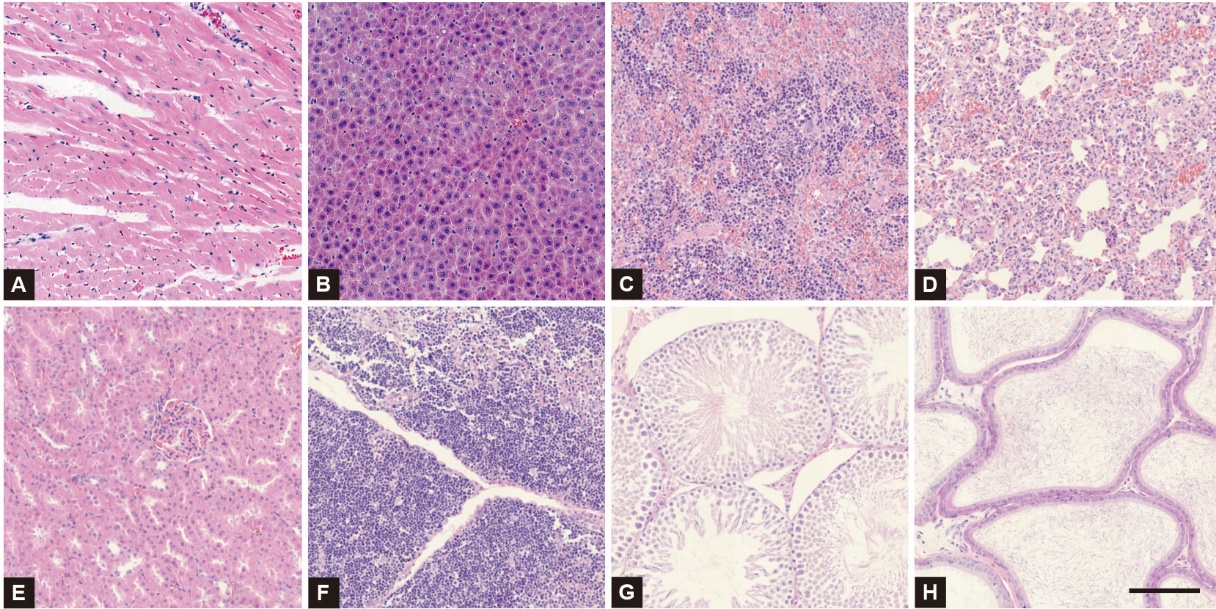


**Supplementary Figure 6**. Histological sections of (A) heart, (B) liver, (C) spleen, (D) lung, (E) kidney, (F) thymus, (G) testis and (H) epididymis of the DM rats implanted with rcBCS after 7 days post-operation. (bar=100μm)


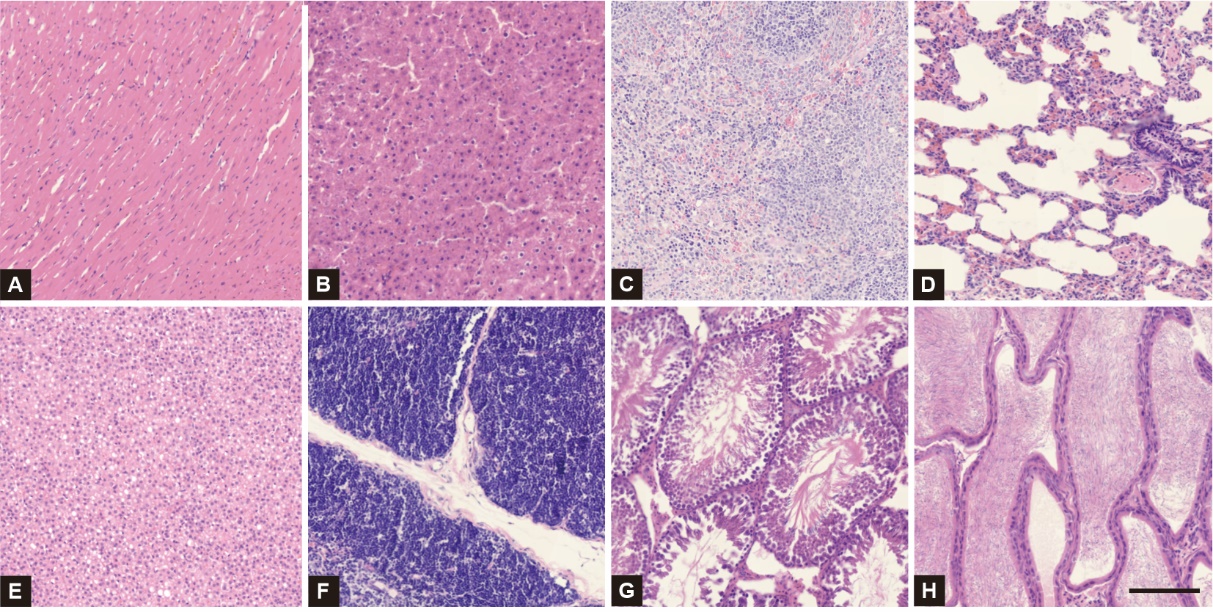


**Supplementary Figure 7**. Histological sections of (A) heart, (B) liver, (C) spleen, (D) lung, (E) kidney, (F) thymus, (G) testis and (H) epididymis of the DM rats implanted with rcBCS after 14 days post-operation. (bar=100μm)


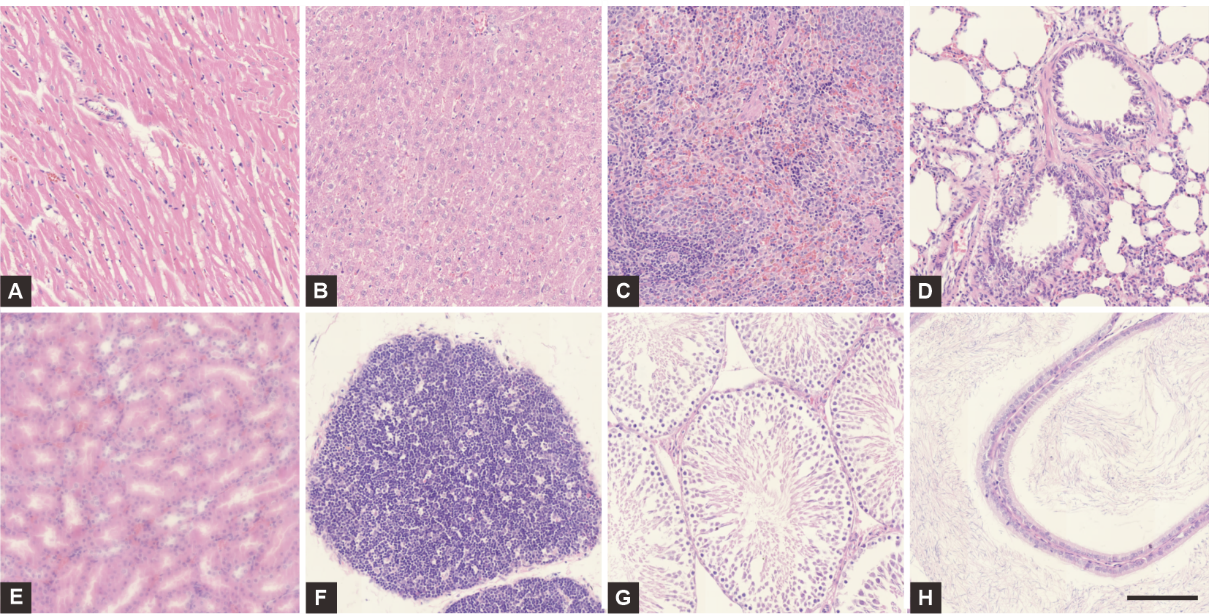


**Supplementary Figure 8.** Histological sections of (A) heart, (B) liver, (C) spleen, (D) lung, (E) kidney, (F) thymus, (G) testis and (H) epididymis of the DM rats implanted with rcBCS after 21 days post-operation. (bar=100μm)


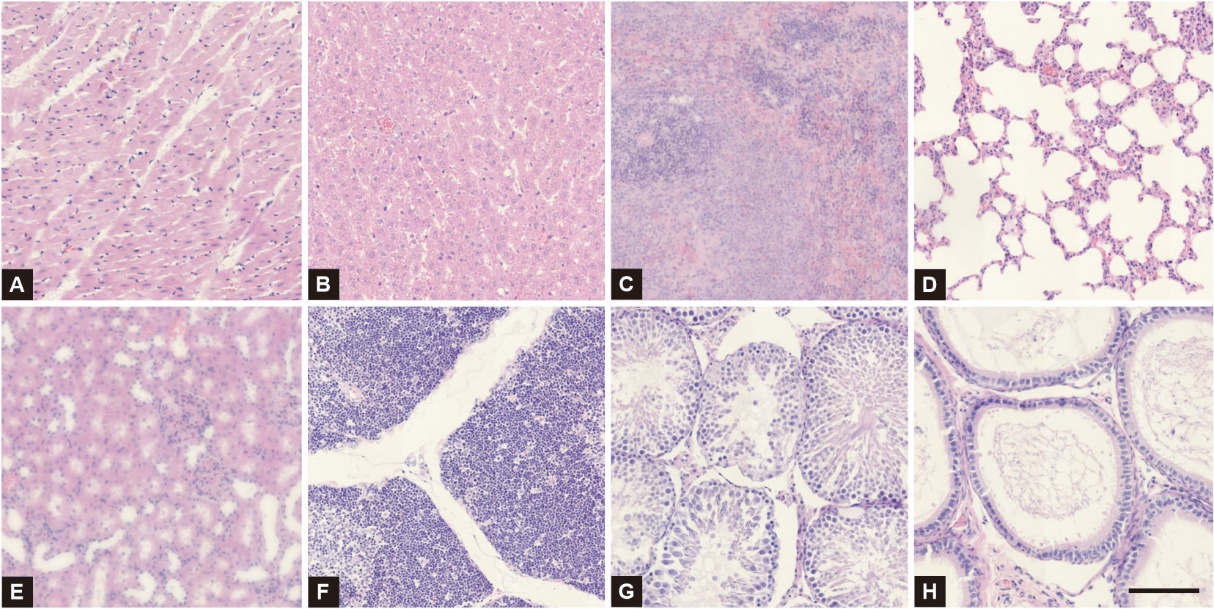


**Supplementary Figure 9**. Histological sections of (A) heart, (B) liver, (C) spleen, (D) lung, (E) kidney, (F) thymus, (G) testis and (H) epididymis of the DM rats in sham operation group after 21 days post-operation. (bar=100μm)

**Reference**:

1 Lou, D., Luo, Y., Pang, Q., Tan, W.-Q. & Ma, L. Gene-activated dermal equivalents to accelerate healing of diabetic chronic wounds by regulating inflammation and promoting angiogenesis. *Bioactive Materials* **5**, 667-679 (2020). <https://doi.org:https://doi.org/10.1016/j.bioactmat.2020.04.018>

2 Huang, Y. Y. *et al.* Effect of a Novel Macrophage-Regulating Drug on Wound Healing in Patients With Diabetic Foot Ulcers: A Randomized Clinical Trial. *JAMA Netw Open* **4**, e2122607 (2021). <https://doi.org:10.1001/jamanetworkopen.2021.22607>

3 Rafat, M. *et al.* Bioengineered corneal tissue for minimally invasive vision restoration in advanced keratoconus in two clinical cohorts. *Nature Biotechnology* **8**, 1-12 (2022). <https://doi.org:https://doi.org/10.1038/s41587-022-01408-w>

4 Zhang, X. *et al.* Biological behavior of fibroblast on contractile collagen hydrogel crosslinked by γ-irradiation. *Journal of Biomedical Materials Research Part A* **102**, 2669-2679 (2014). <https://doi.org:https://doi.org/10.1002/jbm.a.34938>
